# How accurately can we estimate spontaneous body kinematics from video recordings? Effect of movement amplitude on OpenPose accuracy

**DOI:** 10.3758/s13428-024-02546-6

**Published:** 2025-01-02

**Authors:** Atesh Koul, Giacomo Novembre

**Affiliations:** Neuroscience of Perception and Action Lab, Italian Institute of Technology (IIT), Viale Regina Elena 291, 00161 Rome, Italy

**Keywords:** Movement, Motion capture, Marker-less, Computer vision, Kinematics, Video

## Abstract

Estimating how the human body moves in space and time—body kinematics—has important applications for industry, healthcare, and several research fields. Gold-standard methodologies capturing body kinematics are expensive and impractical for naturalistic recordings as they rely on infrared-reflective wearables and bulky instrumentation. To overcome these limitations, several algorithms have been developed to extract body kinematics from plain video recordings. This comes with a drop in accuracy, which however has not been clearly quantified. To fill this knowledge gap, we analysed a dataset comprising 46 human participants exhibiting spontaneous movements of varying amplitude. Body kinematics were estimated using OpenPose (video-based) and Vicon (infrared-based) motion capture systems simultaneously. OpenPose accuracy was assessed using Vicon estimates as ground truth. We report that OpenPose accuracy is overall moderate and varies substantially across participants and body parts. This is explained by variability in movement amplitude. OpenPose estimates are weak for low-amplitude movements. Conversely, large-amplitude movements (i.e., > ~ 10 cm) yield highly accurate estimates. The relationship between accuracy and movement amplitude is not linear (but mostly exponential or power) and relatively robust to camera–body distance. Together, these results dissect the limits of video-based motion capture and provide useful guidelines for future studies.

**Supplementary Information:**

The online version contains supplementary material available at 10.3758/s13428-024-02546-6.

## Introduction

The way the human body moves in time and space—namely, body kinematics—is a rich source of information that is feeding a variety of domains having noteworthy applications for well-being (diagnosis, surveillance), industry (filmmaking, gaming, virtual reality), and research (sport, language, communication) (Colyer et al., 2018; Kidziński et al., 2020; Needham et al., 2021a, 2021b; Parger et al., 2018; Pouw et al., 2020, 2021; Soltani et al., 2016; Toth et al., 2023; Yang Ran et al., 2010).

From a psychological perspective, body kinematics can be seen as a window into humans’ minds or internal states (Becchio et al., 2018). For instance, body kinematics can predict individuals’ intentions (Ansuini et al., 2015a, 2015b; Cavallo et al., 2016; McEllin et al., 2018; Zunino et al., 2017), emotions (Addabbo et al., 2020; de Gelder, 2006; Treal et al., 2020), neurologic disorders, and clinical conditions (Hanakawa et al., 1999; Kidziński et al., 2020; Paolucci et al., 2023; von Schroeder et al., 1995). It follows that interest in motion capture technologies has risen substantially over the past decade (Ansuini et al., 2015a, 2015b; Gowen, 2012).

Currently, the gold standard for precisely measuring body kinematics implies a combination of infrared cameras and reflective markers that are secured on a body of interest. Similar systems—e.g., Vicon, Optitrack, Polhemus—can track the location of a physical marker with excellent temporal (up to millisecond) and spatial (up to a few millimetres) precision (Merriaux et al., 2017; Nagymáté & M. Kiss, 1970; Pouw et al., 2020). Yet, this approach has multiple downsides: it is restrictive (requires multiple cameras and markers), cumbersome, very expensive, and notably not suitable for naturalistic studies or for recordings to be taken out of a laboratory (as might happen in some clinical and research settings or during surveillance; Pouw et al., 2020; Romero et al., 2017; Yang Ran et al., 2010).

To overcome these shortcomings, several video-based motion-tracking technologies have been developed recently (Cao et al., 2017; Mathis et al., 2018). These methods rely solely on video recordings, and therefore are very low-cost, simple to implement, and marker-less. One of such methods—namely OpenPose—relies on deep learning (Cao et al., 2017). Each frame of a video recording is submitted to a pre-trained deep learning model that is capable of inferring the position of a set of ‘virtual’ body markers (or landmarks) associated with specific body parts such as the head, face, torso, arms, hands, legs, and feet. Because this procedure is automatic and does not require any manual labelling from the user or retraining of the underlying model (cf. DeepLabCut Mathis et al., 2018), OpenPose is increasingly being used for human pose detection.

Compared to infrared-based motion tracking, video-based motion tracking such as OpenPose is certainly more accessible and practical. Yet, it yields data with a notably lower temporal and spatial resolution (which is constrained by the low pixel resolution and low frame rate of video camera recordings, compared to infrared recordings). Hence, when choosing to rely on video-based methods, it is important to know whether, and how well, the body kinematics of interest are capturable by this method.

This issue calls for comparative studies that, however, have not reached a consensus about the accuracy of video-based motion capture as compared to infrared-based motion capture. While some studies reported poor or variable accuracy (D’Antonio et al., 2020; Needham et al., 2021a, 2021b; Remedios & Fischer, 2021; Romero et al., 2017), others have reported modest or excellent accuracy (Lin et al., 2022; Martini et al., 2022; Musha et al., 2022; Ota et al., 2020, 2021).

Notably, these previous studies are difficult to compare because they examined (i) movements from different body parts, or (ii) movements having different amplitude, or (iii) recordings taken using heterogeneous perspectives or video equipment. For instance, some of the previous evaluations used movements ranging from squatting, walking in a lab or on a treadmill, up to communicative gestures (Needham et al., 2021a, 2021b; Ota et al., 2020, 2021; Pouw et al., 2020). Therefore, given the variable amount of movement participants would perform under so heterogeneous circumstances, we reasoned that (at least some of) the inconsistencies between these studies might depend on certain factors that might predict the accuracy of video-based body kinematics. Indirectly supporting this reasoning, a recent study has shown how OpenPose mean accuracy changes depending on the specific activity (e.g., walking, running, or jumping) participants are performing (Needham et al., 2021a, 2021b). We thus specifically hypothesized that one of such factors might be the amplitude of the movement of interest.

In the current work, we aimed to assess the impact of movement amplitude on the accuracy of video-based motion-tracking estimates. We examined a dataset including 46 human participants, whose spontaneous body kinematics, having varying amplitude, were recorded using video-based (OpenPose) and infrared-based (Vicon) motion capture systems simultaneously (Koul et al., 2023a, 2023b). Vicon recordings were treated as ground truth. Hence, the two datasets were preprocessed in the same way and reduced to a similar number of body landmarks. Because participants performed spontaneous movements, which typically display unclear movement onsets, we quantified movement amplitude as the body displacement that participants exhibited throughout the course of each trial.

We operationalized the accuracy of OpenPose by computing the degree of correspondence, i.e., the Pearson’s correlation coefficient, between the velocity estimates yielded by the OpenPose and Vicon systems, separately for each body part of interest. We used curve fitting and conditional inference tree analyses to estimate the relationship between OpenPose accuracy and movement amplitude and to generate a model that predicts OpenPose accuracy as a function of progressively larger movement amplitudes. Finally, we evaluated the effect of movement amplitude on OpenPose accuracy as a function of camera–body distance, which we digitally manipulated by increasing it to either 200% or 400% of the original distance. We conclude by providing guidelines for future studies.

## Materials and methods

### Experimental design and procedure

We analysed a previously collected dataset for which 46 individuals (26 female; mean age 21.43 years, range 18–30 years) formed 23 dyads (see Koul et al., 2023a, for more details). The sample size of the original dataset (Koul et al., 2023a) was determined in advance based on previously published studies in the same field of research (Dumas et al., 2010; Goldstein et al., 2018; Hirsch et al., 2017; Noah et al., 2020). This sample size—comprising 46 participants—is much higher than what is typically used for comparisons between infrared and video-based methods (Martini et al., 2022; Nakano et al., 2020; Pouw et al., 2020; Zago et al., 2020). Such evaluations have typically relied on very few—i.e., 2 to 5—participants. The participants forming each dyad were asked to sit face to face, relax, and behave spontaneously. They were not permitted to communicate either verbally or through co-verbal gestures. Participants were generally asked to look at the body of their partner (when this was visible, see below) and not required to necessarily look at each other's faces or eyes. The participants spontaneously produced movements of different body parts including but not restricted to postural adjustments and stretching. They also did not perform any goal-directed movements or manipulate any object (no objects were provided to the participants).

In the original study, two experimental factors were manipulated: visual contact (i.e., whether the participants could see each other or not) and spatial proximity (being either 1 or 3 m). Here, we only analysed the conditions associated with 3 m spatial proximity because those associated with 1 m proximity yielded noisy Vicon estimates (presumably because when the two bodies were too proximal to each other, the visibility of the physical markers was occasionally obstructed). The data analysed here were collected over six repetitions (trials), each lasting 120 s (i.e., 2 min).

### Recordings of body kinematics

#### Video recordings

Video-based recordings were captured by two standalone cameras (SVPRO USB Webcam 5–50 mm varifocal lens, 25 Hz, resolution 1280 × 720) mounted on tripod stands. Each camera recorded body movements from one of the two participants from the front side with a slightly tilted (~ 30°–45° with respect to the participants) aerial and side view, hence leading to two-dimensional (2D) motion capture of each participant (as in Ota et al., 2020, 2021; Pouw et al., 2020). The distance between the cameras and the participants was 316 and 260 cm, respectively. To simultaneously acquire videos from the two cameras, a custom library ‘synchCams’ (https://pypi.org/project/synchCams/) was written in Python. This library allows a frame-locked dual video recording (i.e., the system acquires frames from the two cameras in an alternating fashion). The library utilizes Python-based libraries: ‘opencv’ (https://opencv.org) for video capture, ‘pyserial’ (https://pythonhosted.org/pyserial/) for access to the serial port, and ‘socket’ (https://docs.python.org/3/library/socket.html) for communication over Ethernet.

#### Vicon recordings

**Fig. 1:**
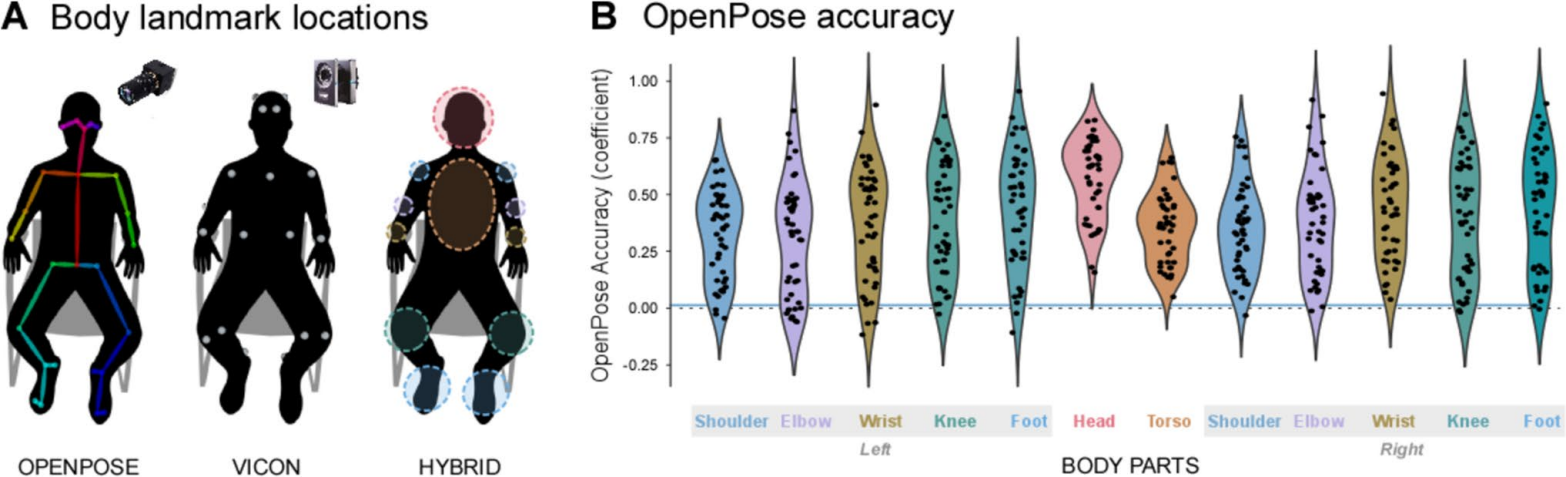
Estimation of body landmarks and accuracy of OpenPose. (**A**) location of body landmarks yielded by OpenPose (left) and Vicon (middle). OpenPose yielded 25 ‘virtual’ body landmarks extracted from the video recordings of each participant. Vicon estimated the position of 18 (for participant 1) and 19 (for participant 2, shown in the figure) wearable retro-reflective markers. The body landmarks/markers yielded by OpenPose and Vicon were further grouped to represent 12 ‘hybrid’ body parts (right) that were comparable across systems (each part is surrounded by a dashed ellipse). (**B**) Violin plots indexing the accuracy of OpenPose [coefficients resulting from the Pearson’s correlation between movement velocity timeseries yielded by OpenPose and Vicon (ground truth). Coefficients were computed separately for each trial, body part, and participant (dots)]. Relatively higher positive coefficients index relatively higher accuracy, while coefficients at zero (dashed line) or lower (negative) index inaccurate estimates (the blue horizontal line indexes chance accuracy following a non-parametric permutation analysis). Note that OpenPose accuracy was overall variable: it changed considerably across distinct participants and body parts

Marker-based three-dimensional (3D) recordings were acquired using a system of eight near-infrared cameras (frame rate: 250 Hz; Vicon System), recording the two bodies from 1.5–2 m distance. Participants wore light-weight retro-reflective hemispheric markers (participant 1: 18 markers, participant 2: 19 markers; see Fig. 1A) across multiple body parts: head (participant 1: 3 markers, participant 2: 4 markers), torso (3 markers), left shoulder (1 marker), left elbow (1 marker), left wrist (1 marker), right shoulder (1 marker), right elbow (1 marker), right wrist (1 marker), left knee (2 markers), left foot (1 marker), right knee (2 markers), right foot (1 marker) (see Fig. 1A). This marker-based motion capture system generated 3D kinematics data in contrast to the 2D data obtained from videos.

### Data analysis

#### Preprocessing of video-based kinematics

We estimated the position of landmarks (i.e., virtual markers) from the video recordings using OpenPose, an automated machine learning-based algorithm (Cao et al., 2017). Single frames from the videos were submitted to a pre-trained multi-stage convolutional neural network (CNN). The network jointly predicts part affinity fields (encoding location and orientation of limbs in the image domain) and confidence maps for body part detection. Subsequently, a greedy inference parsing the confidence maps yielded body and hand landmarks. A total of 25 body landmarks (including head, torso, arms, and legs) were estimated. The pre-trained model was loaded using a custom library (pytorch_openpose) implementing the OpenPose Python application programming interface (Cao et al., 2017). Estimation of the landmarks was performed over an NVIDIA GeForce RTX 2060 SUPER graphics processing unit (GPU).

The predicted landmarks convey 2D locations (relative to the video field of view with the origin at the top left corner of the image) of anatomical body parts. The data were preprocessed as in the original study for which the data were collected (cf. Koul et al., 2023a). Specifically, we first removed outlying values (3 standard deviations away from the mean computed within each trial, 0.68% of all data) and data points where the algorithm failed to predict body position (8.0% of all data) (supplementary Figure S1). The data timeseries were then interpolated using a one-dimensional (1D) interpolation (table lookup) and smoothed using a moving mean (window size = 1 s). Movement velocity was computed by taking the first derivative of the positional data along the *x* and *y* axes separately and then calculating the Euclidean norm of the resulting velocity vectors. This velocity measure estimates body part displacement over time irrespective of the spatial direction of the movement (similar to computing movement speed). These new timeseries were also smoothed by applying a moving mean window of 1 s. The timeseries were further normalized (*z*-scoring) separately for each body part and down-sampled to 10 Hz [cf. Nakano et al., 2020; Ota et al., 2020; Mehdizadeh et al., 2021; Washabaugh et al., 2022; for control purposes, we also checked whether a different sampling (25 Hz) would lead to different results, but this was not the case as we show in supplementary Figure S2]. To exclude the presence of movement artifacts (i.e., high degree of variance), the data were visually inspected, and 2.17% of the timepoints were manually excluded from further analyses (supplementary Figure S3). Finally, to make this data comparable with that collected with Vicon, the 25 body landmarks were grouped to represent 12 different body parts: head (mean of 5 landmarks), torso (mean of 4 landmarks), left shoulder (1 landmark), left elbow (1 landmark), left wrist (1 landmark), right shoulder (1 landmark), right elbow (1 landmark), right wrist (1 landmark), left knee (1 landmark), right knee (1 landmark), left foot (mean of 4 landmarks), and right foot (mean of 4 landmarks) as shown in Fig. 1A (hybrid).

We decided to use velocity profiles instead of positional data for two main reasons. First, many previous studies assessing the accuracy of OpenPose have used velocity (e.g., Needham et al., 2021a, 2021b; Vieira et al., 2022; Cronin et al., 2024). Secondly, some studies have explicitly recommended the use of velocity as an evaluation measure, possibly because numerical differentiation, as compared to positional time courses, leads to stronger sensitivity to potential mismatch between video-based and ground truth estimates (Seethapathi et al., 2019; Mehdizadeh et al., 2021; Cronin, 2021).

#### Preprocessing of Vicon-based kinematics

Each trial was visually inspected to verify correct marker identification as well as to identify missing data points. When missing data points were identified, these were interpolated using the ‘Manually fill gaps’ pipeline from Vicon Nexus. The algorithms used were either ‘Pattern fill’ (relying on the trajectory of the closest marker on the same body part) or ‘Rigid body fill’ (when a rigid or semi-rigid relationship existed between the markers). The data were then exported, in the form of 3D landmarks (*x*, *y*, and *z*), down-sampled to 10 Hz (e.g., Nakano et al., 2020), and preprocessed following the same pipeline described above in relation to the video-based data.

### Manipulating camera–body distance

We digitally manipulated the distance between the cameras and the participants to assess its impact on OpenPose accuracy. For this, we scaled the original images to either 50% or 25% of their original size, while preserving the original image resolution (thus increasing the camera–body distance to 200% and 400% respectively). Consequently, the resolution of the images remained the same (i.e., 1280 × 720 pixels), while the participants’ size was scaled by 50% or 25%. The new scaled images still had the participant in the middle of the image, while values of 0 were added on the borders. OpenPose was used again to predict body landmarks from these new videos (as done above).

### Statistical analyses

#### Estimation of OpenPose accuracy

OpenPose accuracy was estimated using Pearson’s correlations. Specifically, we correlated pairs of movement velocity timeseries, yielded by OpenPose (2D time series) and Vicon (3D time series serving as ground truth), capturing a given participant, body part, and trial (see supplementary Figure S4 for representative trials). Had we computed such correlations using two sets of 3D data, then the accuracy of our estimates would have probably been higher, but we have no reason to assume that this would have changed our results (if not improved them). Prior to computing the coefficients, we did not segment the velocity timeseries in order to capture individual movements because, in the context of spontaneous behaviour, multiple individual movements might overlap in time, making it difficult to identify clear boundaries. The resulting coefficients were averaged across trials to obtain one correlation coefficient for each participant and body part. To test whether these correlations were significantly different from chance level, we performed one-sample *t*-tests comparing the resulting coefficients (one per participant and body part) versus 0, which was assumed to signify chance level. To validate this assumption, we also performed a non-parametric permutation-based test. For this analysis, we computed surrogate correlation coefficients between the Vicon time series associated to one body part in a given trial *n* and OpenPose timeseries associated to the same body part for all trials but trial *n* (leading to surrogate trials). We repeated this procedure across all trials, participants, and body parts. We then averaged the resulting coefficients across trials, body parts, and participants to obtain an average ‘surrogate’ correlation coefficient. To test whether the correlation coefficients were different across body parts, we submitted the coefficients to a one-way repeated measures analysis of variance (ANOVA) with ‘Body parts’ as main factor. In case of violations of sphericity, we applied a Greenhouse–Geisser correction to the degrees of freedom. Post-hoc tests were corrected for multiple comparisons using Holm–Bonferroni correction.

#### Relationship between OpenPose accuracy and movement amplitude

Using two distinct analyses, we examined to what extent OpenPose accuracy depended on the amplitude of the spontaneously generated movements. Movement amplitude was chosen because it is a simple movement parameter to observe and to quantify. However, other (cor)related parameters could be used (e.g., in supplementary Figure S5, we show how accuracy also depends on movement velocity besides movement amplitude). Movement amplitude was operationalized as the displacement of each body part throughout the course of each trial, and it was assessed using the ground truth Vicon data (sampled at 250 Hz). Specifically, displacement values (at trial-by-trial level) were estimated as the range (max value – min value) of the Euclidean distance vector between the origin (located at the floor of the experimental room) and the *x*, *y*, and *z* coordinate at each time point. These amplitude values were then averaged across trials to get one value for each body part and participant.

In the first analysis, we used ‘curve fitting’ to estimate the mathematical function that best described how accuracy changed with movement amplitude at participant level, separately for each body part. The OpenPose accuracy values (coefficients) were plotted against their associated movement amplitude values, separately for each body part. Next, we used the MATLAB Curve Fitting Toolbox to fit six different functions: linear, quadratic, exponential (1 and 2 terms), and power (1 and 2 terms) (Table 1). The fit of the functions was assessed using adjusted *r*-squared.

**Table 1:** Curve-fitting algorithms and their respective mathematical functions

Curve type	Function
Linear	*f*(*x*) = *p*1 × *x* + *c*
Quadratic	*f*(*x*) = *p*1 × *x*^2 + *p*2 × *x* + *c*
Exponential single term	*f*(*x*) = *a* × exp(*b* × *x*)
Exponential two terms	*f*(*x*) = *a* × exp(*b* × *x*) + *d* × exp(*e* × *x*)
Power single term	*f*(*x*) = *a* × *x*^*b*
Power two terms	*f*(*x*) = *a* × *x*^*b* + *c*

Where *p1, p2, a,* and *d* are function coefficients, while *c* is a constant term

In a second analysis, we generated a predictive model using a conditional inference tree (CIT). This model was meant to predict OpenPose accuracy from movement amplitude. CIT is a non-parametric class of decision trees that constructs predictive models by recursively partitioning (splitting) the dataset based on statistical hypothesis testing (Hastie et al., 2009; Hothorn et al., 2006; Loh, 2011). The splits can be represented as a tree that stops growing when the statistical tests are no longer significant. CITs produce models that are interpretable and, compared to other decision tree algorithms (e.g., classification and regression trees), they are less prone to overfitting. We generated the CIT using correlation coefficients (averaged over trials) from all the body parts and participants, and predicted OpenPose accuracy using movement amplitude. We modelled all the body parts together to ensure that the analysis had an adequate amount of training data (cf. Van Der Ploeg et al., 2014). This is important because machine learning algorithms require a large number of data points to yield reliable estimates (Hastie et al., 2009; Van Der Ploeg et al., 2014). For instance, for tree-based models such as the one we used here, stable estimates are generated when using more than 62 samples per variable (Van Der Ploeg et al., 2014). In our case, estimating the CIT for each body part would have led to unreliable training based on only 46 data points. To evaluate the performance of our approach, we used a leave-one-subject-out cross-validation (Koul et al., 2018). All CIT analyses were conducted using the ‘PredPsych’ library (Koul et al., 2017).

#### Impact of distance and movement amplitude on OpenPose accuracy

In this analysis, we estimated the impact of distance (between the body of interest and the cameras) and movement amplitude on OpenPose accuracy. To do this, we split our data (participant movement amplitude averaged at trial and body part level) to form three tertiles: each including the participants displaying the lowest, medium, or largest movement amplitude throughout the experiment. Together, this and the camera–body distance manipulation yielded a 3 × 3 factorial design with distance (original distance, 200% distance, and 400% distance) and movement amplitude (low, medium, and high movement amplitude) as factors. We used a linear mixed-effects analysis to estimate the effects of distance and movement amplitude.

## Results

OpenPose accuracy was indexed by coefficients resulting from the Pearson’s correlation between OpenPose and Vicon (ground truth) velocity estimates. In what follows, coefficients (absolute *r* values) < 0.10 are regarded as indicative of OpenPose being ‘inaccurate’. Accuracy was regarded as ‘weak’ when the coefficients ranged between 0.10 and 0.39, as ‘moderate’ when the coefficients ranged between 0.40 and 0.69, as ‘strong’ when the coefficients ranged between 0.70 and 0.89, and finally as ‘perfect’ when the coefficients were higher than 0.90. These are of course arbitrary limits, but at least they provide objective definitions of the labels we use throughout the manuscript to assess accuracy. A similar classification has been used previously (Akoglu, 2018; Dancey & Reidy, 2011).

### OpenPose accuracy in estimating body kinematics

Overall, OpenPose accuracy in estimating body kinematics of individual participants varied from being inaccurate (coefficient = − 0.12) to being perfect (coefficient = 0.96) (see Fig. 1B, dots represent individual participants; see also supplementary Figure S4 for examples of raw time courses). Averaging participants’ estimates, accuracy was moderate (0.40), and it varied considerably across different body parts (from 0.31 to 0.57).

Statistics on these data indicated that the coefficients were significantly positive (i.e., they were higher than zero correlation coefficient indexing ‘chance’ accuracy) for all body parts [*p*s < 0.001; Cohen’s *d* ranging from 1.23 (left elbow) to 3.32 (head)]. This result was also confirmed by a non-parametric permutation-based analysis for which we compared the coefficients with a ‘chance’ value generated from surrogate data (*p*s < 0.05 FDR corrected). Further, an ANOVA on these data yielded a significant effect of 'Body parts’ (*F*(11, 484) = 16.8, *p* < 0.001, generalized η^2^ = 0.083), indicating that the overall accuracy (i.e., averaged across participants) ranged from being moderate for body parts such as the head (median coefficient = 0.63) to weak–moderate for other body parts such as the wrists [median coefficient = 0.40 (left) and 0.44 (right)], the feet [median coefficients = 0.48 (left) and 0.50 (right)], the shoulders [median coefficients = 0.36 (left) and 0.34 (right)], the elbows [median coefficients = 0.34 (left) and 0.37 (right)] and the torso (median coefficient = 0.37). We also confirmed these results using intraclass correlation coefficients instead of Pearson’s correlation coefficients (supplementary Figure S6).

That overall accuracy varied significantly across different body parts might be partly explained by the fact that some body parts were more likely than others to display large-amplitude movements, either due to biomechanical properties or to task constraints (i.e., movements were recorded while participants were sitting and therefore were not able to freely move all body parts equally). In the next section, we explicitly test whether movement amplitude predicts OpenPose accuracy.

### OpenPose accuracy depends on movement amplitude

We formally tested (i) whether OpenPose accuracy depended on movement amplitude and (ii) whether such a putative relationship would be best described by a linear or non-linear (i.e., quadratic, power, or exponential) function.

**Fig. 2:**
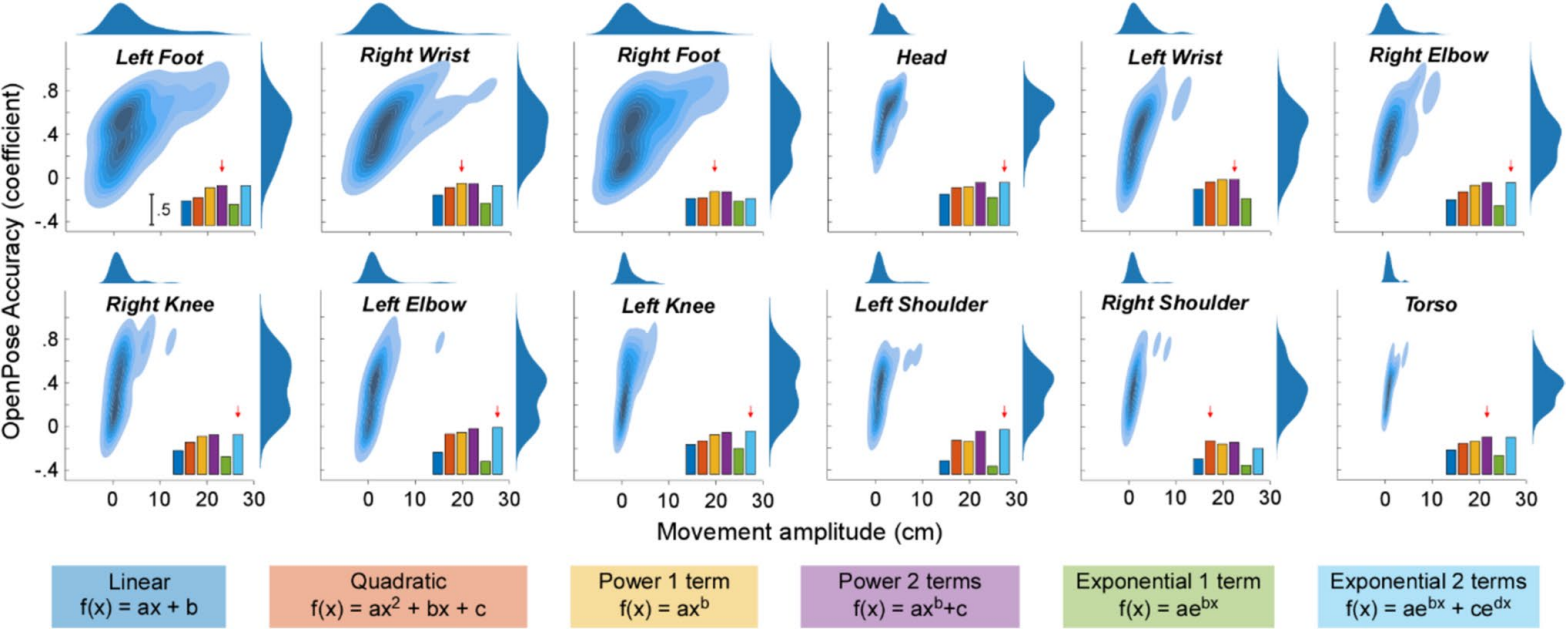
Relationship between OpenPose accuracy and movement amplitude. For each of the 12 body parts, plots represent both data density and relationship between OpenPose accuracy (coefficient, y-axis) and amplitude of movement (in cm, x-axis). The kernel density estimate (KDE; blue clouds in the middle of all plots) reflects the probability density of the relationship between the OpenPose accuracy and the movement amplitude. Given the scatter plot of these two variables, KDE represents the overall pattern of the data, showing where there are more points clustered together and where they are more spread out. It helps showing the distribution of data by creating a smooth curve that represents how likely it is to find a data point at any given point along the curve. Darker colours represent higher probability while lighter colours represent lower probabilities. The distributions on the top and to the right of each plot show the distribution of each variable (independently of their relationship). These distributions help visualizing whether the variables are normally distributed, which is a requirement for computing Pearson’s correlation and for most of the statistical tests performed. Insets of each plot show the results of curve fitting (adjusted *r*-squared) for six different functions (best-fitting functions indicated by red arrows). Non-linear functions (power and exponential) typically showed higher *r*-squared values than a linear function (dark blue bars). Plots are arranged in a descending order according to movement amplitude i.e., body parts with higher movement amplitude are plotted first. For left wrist, the adjusted *r*-squared for the exponential 2 terms function is not plotted as it generated a negative goodness of fit

Figure 2 displays density plots for the relationship between OpenPose accuracy (i.e., correlation coefficients) and movement amplitude (density computed at participant level). With increasing movement amplitude, OpenPose accuracy clearly grew from being very weak to being very strong or perfect. This was so for all body parts.

Figure 2 also displays the goodness of the curve fit (adjusted *r*-squared) for six different functions (see bar plots in Fig. 2, and Table 1 above). The results from this analysis indicated that OpenPose accuracy increases non-linearly with movement amplitude (adjusted *r*-squared for best-fitting functions ranged from 0.53 to 0.75). Indeed, exponential, power, and quadratic relationships were better predictors than linear ones. Specifically, Exponential 2 terms function yielded the best fit of the data in 50% of the cases (six of 12 body parts), followed by Power 2 terms (~ 25%, 3 out of 12 body parts), then Power 1 term (~ 16.67%, 2 out of 12 body parts), and quadratic functions (~ 8.33%, 1 out of 12 body parts).

Together, these results indicate that OpenPose accuracy grows with movement amplitude and that the relationship between accuracy and amplitude is not linear.

### Modelling the relationship between OpenPose accuracy and movement amplitude

Having established a non-linear relationship between OpenPose accuracy and movement amplitude, we modelled such relationship using a conditional inference tree. The results from this analysis were corroborated by a leave-one-subject-out cross-validation yielding a low error (average mean squared error = 0.023; SEM = 0.0024).

**Fig. 3:**
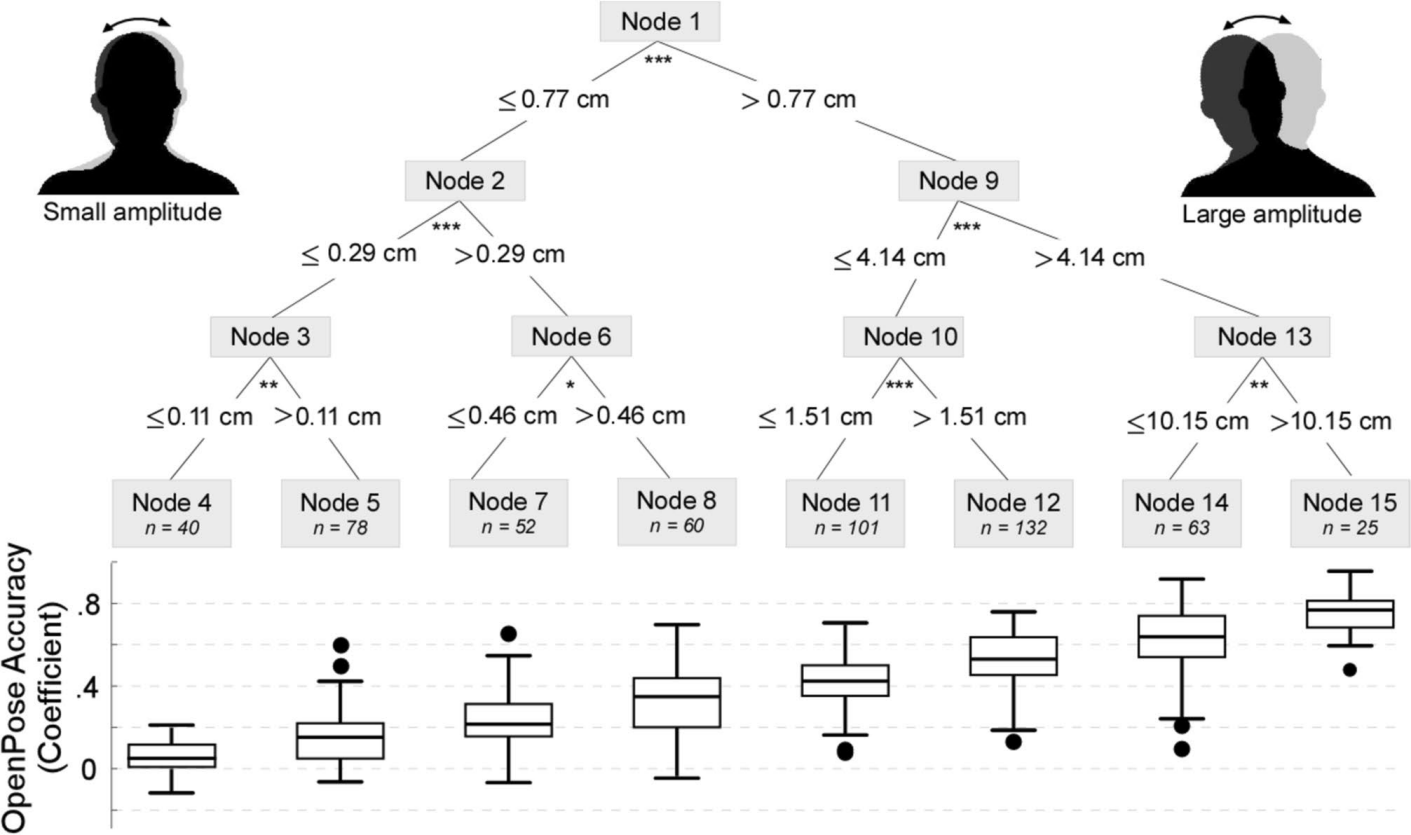
Condition inference tree modelling the relationship between OpenPose accuracy and movement amplitude (all body parts combined). The tree predicts OpenPose accuracy using a set of conditional statements (if–then rules). The results from this analysis show that when movement amplitude is lower than e.g., 0.29 cm (node 3), OpenPose accuracy is very weak (mean coefficient = 0.12). Conversely, when movement amplitude is more than e.g., 10.15 cm (node 15), then OpenPose accuracy is strong (mean coefficient = 0.75). Several other nodes are yielded. These other nodes are non-linearly distributed in between 0.11 cm and 10.15 cm, and their associated accuracy is reported. All nodes in the tree were significant (i.e., the splits explained significant variance). *N* represents the number of data points associated with the end nodes (the ones plotted at the bottom). * *p* < 0.05, ** *p* < 0.01, *** *p* < 0.001

The resulting model (trained on all participants and body parts) is displayed in Fig. 3. The model yielded 15 non-linearly distributed nodes, leading to eight movement amplitude intervals (ranging from small to large scale) that predicted increasing levels of OpenPose accuracy. Specifically, OpenPose was weak (mean coefficient = 0.12) when movement amplitude was lower than 0.29 cm (nodes 4 and 5). Accuracy grew but remained weak with movement amplitudes between 0.29 cm and 0.46 cm (mean coefficient = 0.24) and between 0.46 cm and 1.51 cm (mean coefficient = 0.39). It increased from weak to modest with movement amplitudes between 1.51 cm and 10.15 cm (mean coefficient = 0.56). Finally, accuracy was strong (mean coefficient = 0.75) when movement amplitude was larger than 10.15 cm (maximum recorded movement amplitude = 24.70 cm) (node 15).

Together, this analysis provides a very detailed estimation of how accuracy grows with increasing movement amplitude, at least in our recording context.

### OpenPose accuracy depends on both camera–body distance and movement amplitude

Having assessed a robust relationship between OpenPose accuracy and movement amplitude, we next assessed whether this relationship might also manifest itself when changing the spatial distance between the camera and the body of interest.

**Fig. 4:**
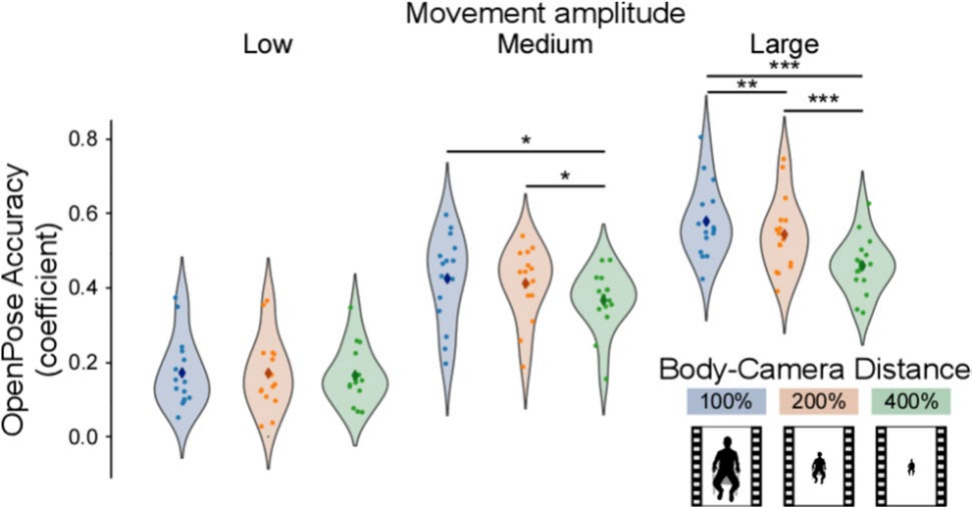
Impact of camera–body distance and movement amplitude on OpenPose accuracy. Violin plots indexing OpenPose accuracy as a function of camera–body distance and movement amplitude. OpenPose accuracy grew with movement amplitude. Additionally, with increasing distance, accuracy dropped only for large- and medium-amplitude movements. * *p* < 0.05, ** *p* < 0.01, *** *p* < 0.001

To capture variations across multiple levels (participants, body parts, spatial distance), we performed a linear mixed-effects analysis. Based on our hypotheses, we compared two models: Model 1 with ‘Distance’, ‘Movement amplitude’, and ‘Body parts’ and Model 2 with ‘Distance’ and ‘Movement amplitude’ as fixed effects. We estimated by-subject (random effect) intercepts and slopes for ‘Body parts’ and ‘Distance’. The results of this analysis show that Model 2 performs better [lower Bayesian information criterion (BIC)] than Model 1 (Model 1 BIC = − 1870.2; Model 2 BIC = − 2288.1). Investigating the effects of Model 2, we report a significant effect of ‘Movement amplitude’ (χ^2^(2) = 106.57, *p* < 0.001) and a significant ‘Distance’ × ‘Movement amplitude’ interaction (Fig. 4; χ^2^(4) = 35.67, *p* < 0.001). Specifically, as low movement amplitude was associated with inaccuracy or weak accuracy, these estimates did not change as a function of distance (Bonferroni-corrected *p* values for low movement movements: 100% vs 200% *p* = 1.0, mean difference = 0.001, 95% CI = [− 0.017, 0.019]; 100% vs 400% *p* = 1.00, mean difference = 0.007, 95% CI = [− 0.02, 0.034]; 200% vs. 400% *p* = 1.00, mean difference = 0.006, 95% CI = [− 0.02, 0.032]). Instead, medium and high movement amplitudes, which were associated with weak-to-moderate and moderate-to-strong accuracy, respectively, dropped significantly with increasing spatial distance (Bonferroni-corrected *p* values for medium movement—100% vs 200% *p* = 1.0, mean difference = 0.013, 95% CI = [− 0.005, 0.031], 100% vs 400% *p* = 0.004, mean difference = 0.058, 95% CI = [0.031, 0.086]; 200% vs 400% *p* = 0.041, mean difference = 0.045, 95% CI = [0.02, 0.071]; Bonferroni-corrected *p* values for high movement amplitudes—100% vs 200% *p* = 0.008, mean difference = 0.035, 95% CI = [0.018, 0.052], 100% vs 400% *p* < 0.001, mean difference = 0.12, 95% CI = [0.092, 0.145], 200% vs 400% *p* < 0.001, mean difference = 0.084, 95% CI = [0.059, 0.11]). This drop in accuracy was generally not very pronounced, indicating that accuracy was relatively robust to camera–body distance.

Together, these results indicate that camera–body distance is a moderate predictor of OpenPose accuracy, but only when movement amplitude is sufficiently high. With low movement amplitude, OpenPose is inaccurate, and therefore, the interaction with distance is negligible.

## Discussion

In the current study, we investigated OpenPose accuracy in estimating body kinematics. We estimated movement velocities from body landmarks predicted by OpenPose and compared them to analogous measures yielded by Vicon (ground truth). We show that OpenPose is overall moderately accurate in estimating body kinematics and that this estimation varies significantly across participants and body parts. We then tested whether this variability reflected the variable amplitude of the measured movements. We found that indeed, OpenPose accuracy depends on movement amplitude, with strong accurate estimates being selectively associated with relatively large-amplitude movements (i.e., > 10.15 cm). Finally, we show that the relationship between OpenPose accuracy and movement amplitude is non-linear, and it is relatively robust to camera–body distance.

### OpenPose accuracy depends on movement amplitude

The variation in OpenPose accuracy across different body parts and participants could reflect the fact that participants in our study performed spontaneous body movements. The spontaneity of the movements measured in our study—as opposed to instructed movements most studies look at (Nakano et al., 2020; Needham et al., 2021a, 2021b; Ota et al., 2020)—allowed us to capture a large variability in movement amplitude. This is something that prior studies could not exploit because when participants perform an instructed (often repetitive) task, spontaneous variability is suppressed (Lafe & Newell, 2022; Lai et al., 2015).

The amplitude of these spontaneous movements varied both across participants and body parts, and this impacted upon OpenPose accuracy. We used six different functions to formalize the relationship between movement amplitude and OpenPose accuracy using a curve-fitting procedure. We demonstrated that OpenPose accuracy grew non-linearly with movement amplitude. Specifically, exponential and power functions best fit this relationship. These best-fit functions had a steep initial rise profile that flattened (plateaued) towards the end. This profile suggests that OpenPose accuracy increases rapidly when movements start becoming noticeable and reaches a ceiling level after which accuracy no longer grows significantly. This level might vary across body parts. For instance, accuracy in estimating the left foot grew sharply [from inaccurate (− 0.11) to weak (0.37)] when amplitudes changed from 0 to 5 cm, and less so [from weak (0.37) to moderate (0.64)] when amplitudes changed from 5 to 21.12 cm (Fig. 2). Instead, the accuracy for right knee grew sharply from being inaccurate (− 0.02) to weak (0.34) when the amplitude changed from 0 to 3 cm, and less so [from weak (0.34) to strong (0.73)] when the amplitude changed from 3 to 12.32 cm.

We modelled the relationship between OpenPose accuracy and movement amplitude using a conditional inference tree, now taking all body parts into account. The results of this analysis confirm that OpenPose shows a significant improvement in accuracy as the amplitude of movement increases, following a non-linear relationship similar to that illustrated above. This analysis, notably relying on a data-driven approach, further indicated that accuracy becomes strong once movement amplitude exceeded ~ 10 cm. Of course, this specific threshold might change across different recording settings, as it might also depend on the nature of the captured behaviour or participants’ posture. However, our findings demonstrate how OpenPose performs very differently across frames picturing movements of varying amplitude. This further implies that body parts exhibiting relatively low-amplitude movements, such as the shoulder or the elbow if participants are sitting as in our study, will generally be associated with the lowest kinematics accuracy. Similarly, OpenPose will perform relatively poorly when estimating the position of body parts that can move relatively less due to biomechanical constraints. For instance, movements like shoulder hyperextension and adduction (whose movement range is around 50°; Luttgens & Hamilton, 1997) could lead to inaccurate to weak accuracy (for an average upper arm length of 36 cm). In contrast, movements like shoulder flexion and extension (whose movement range is around 180°; Luttgens & Hamilton, 1997) might lead to better predictions.

Our finding carries significant implications for prior research in the field. Previous studies have suggested that, under certain conditions, OpenPose would be inaccurate because of shortcomings of the algorithm (Needham et al., 2021a, 2021b; Palucci Vieira et al., 2022; Stenum et al., 2021). However, our findings question these interpretations by demonstrating that the accuracy of OpenPose crucially depends on variations in spontaneous movement amplitude, something that previous studies did not consider. This might explain the stark contrast between some previous reports. For instance, previous investigations have reported low accuracies associated with certain body parts such as the hip and knee (D’Antonio et al., 2020; Needham et al., 2021a, 2021b). Was this due to algorithmic limitations or to the small-scale amplitude of the captured movements? It is plausible that discrepancies in accuracy estimates reported across studies are explainable by cross-study variability in movement amplitude.

In the current study, we focused on movement amplitude because it is a simple movement parameter to observe and to quantify. However, we acknowledge that the above should not be taken as to imply that movement amplitude is the *only* parameter that can predict OpenPose accuracy. This is indeed unlikely. As movement amplitude is (cor)related to several (also informative) movement parameters, we do not claim this is the only informative one. On the contrary, we have shown that another movement parameter, specifically movement velocity, can predict OpenPose accuracy, although this relationship is best characterized by a different (i.e., quadratic) mathematical function (see supplementary Figure S5). This implies that, while large-amplitude movements normally lead to higher OpenPose accuracy, the same might not hold true for large velocity movements, which instead might occasionally yield relatively imprecise estimates. The precise relationship between these parameters, and their impact upon video-based motion capture accuracy, could be further explored in the future.

### OpenPose is relatively robust to camera–body distance.

Having shown a significant relationship between movement amplitude and OpenPose accuracy, we investigated how this relationship changes as a function of camera–body distance. Our results revealed a significant reduction in OpenPose accuracy as the camera–body distance increased, but only associated with medium and large amplitude body movements. When quadruplicating the distance (from 100 to 400%), the accuracy of the estimates associated with medium and large amplitude body movements dropped by 14% and 21%, respectively. Instead, accuracy of low-amplitude movements was not affected by increasing camera–body distance. The latter finding might be explained by the fact that low-amplitude movements are originally estimated poorly, and therefore their accuracy cannot be further compromised.

Previously, distance manipulations have been presumed to have a low impact on OpenPose accuracy, although this was not empirically tested (Nakano et al., 2020). Further, there are no formal guidelines or recommendations for optimal camera–body distance (Kim et al., 2021). Perhaps this is because OpenPose accuracy is not particularly dependent on such distance, as our results indeed suggest. Regardless, our results provide a quantitative estimation of the relative robustness of OpenPose, at least across the specific distances we examined. Increasing camera–body distance might only lead to a moderate decrease in OpenPose accuracy, suggesting that it might be a less important factor (compared to movement amplitude) to consider while setting up video-based analyses.

### Implications for future studies

The current work has implications for future studies across three distinct domains. First, it can serve as a benchmark for future investigations that aim to estimate body kinematics in a low-cost, simple-to-implement, and marker-less manner. Our work informs researchers on whether they can adequately substitute traditional infrared-based approaches with video-based approaches, specifically OpenPose. More importantly, depending upon the anticipated range of movement amplitude and relative camera–body distance, researchers can get an estimate of how accurately OpenPose will capture body kinematics. For instance, if the researchers are interested in body movements that are around 4–10 cm in amplitude (range of wrist movement in typical tasks like drinking, pouring, and eating), they can expect video-based recordings to capture approximately 62% of the variance captured by traditional infrared-based recordings. This suggests that using OpenPose for tracking such movements can lead to a loss of 38% of variance compared to a traditional infrared system. Conversely, studies that use OpenPose to capture lower-amplitude movements (e.g., manipulating small objects with fingers) that might range between 0.5 and 1 cm would be able to capture only ~ 36% of the variance capturable by infrared-based recordings. This would lead to notably lower accuracies. Thus, OpenPose might not be useful in tracking kinematics in such studies. These estimates might need to be confirmed by other studies and under different recording conditions.

Second, our results are relevant for studies comparing the accuracy of OpenPose with other video- and non-video-based motion capture systems. We highlight the importance of considering factors such as movement amplitude (or velocity) and camera–body distance when performing these evaluations. This also implies that studies in this field should only be compared when looking at the same body part and same task (e.g., running, squatting) because these can be intrinsically linked with variable levels of amplitude. Without adequately considering these factors, it becomes difficult to discern whether low accuracy stems from inherent algorithm limitations or whether it is simply due to the low amplitude of the movements performed. Therefore, our findings strongly advocate for incorporating movement amplitude as a key metric in the evaluation and assessment of not only OpenPose but also other video-based approaches such as mediapipe (Bazarevsky et al., 2020) and Alphapose (Fang et al., 2022). This will lead to a more comprehensive understanding of the algorithm's performance and its applicability in various contexts.

Finally, our study further highlights the need for better algorithms and datasets that focus on estimating kinematics parameters (such as velocities, acceleration) rather than only estimations of pose (i.e., landmark locations). Indeed, for most applications, kinematic parameters are the main parameters of interest (Cronin, 2021; Mehdizadeh et al., 2021; Seethapathi et al., 2019). In our current study, we find that the OpenPose tracks poorly or is unable to track body velocity, especially when associated with low-amplitude movements. One of the reasons for such failure could be that the training dataset for OpenPose (like other currently available neural networks) entails single images rather than continuous videos. Thus, OpenPose is not trained specifically on body kinematics data but performs pose estimation independently on every single videoframe. Future endeavours might develop algorithms that take into account the evolution of body kinematics over time and/or are trained on such kinematic datasets.

## Supplementary Information

Below is the link to the electronic supplementary material.

Supplementary file1 (PDF 793 kb)

## Data Availability

Raw data that support the findings of this study are available in IIT dataverse repository: 10.48557/SGLTVT

## Code availability

Data analysis libraries and scripts are available on GitHub: https://github.com/ateshkoul/OpenPose_evaluation

## Declarations

### Open practices statement

Materials and analysis code are available in trusted repositories.
