# Supplementary material for: How accurately can we estimate spontaneous body kinematics from video recordings? Effect of movement amplitude on OpenPose accuracy
Source: Behav Res Methods. 2025 Jan 2;57(1):38. doi: 10.3758/s13428-024-02546-6 (PMC11695451; doi:10.3758/s13428-024-02546-6)
Supplement: Supplementary file 1 — Supplementary file1 (PDF 793 kb) [file 13428_2024_2546_MOESM1_ESM.pdf]

Supplementary information for

How accurately can we estimate spontaneous body kinematics from video recordings? Effect of movement amplitude on OpenPose accuracy

Atesh Koul[1*] & Giacomo Novembre[1*]

[1] Neuroscience of Perception and Action Lab, Italian Institute of Technology, Rome, Italy.

*Correspondence to: Atesh Koul and Giacomo Novembre

Italian Institute of Technology (IIT),

Viale Regina Elena 291, 00161 Rome, Italy

Email: atesh.koul@iit.it, giacomo.novembre@iit.it

Phone: +39 06 49255199

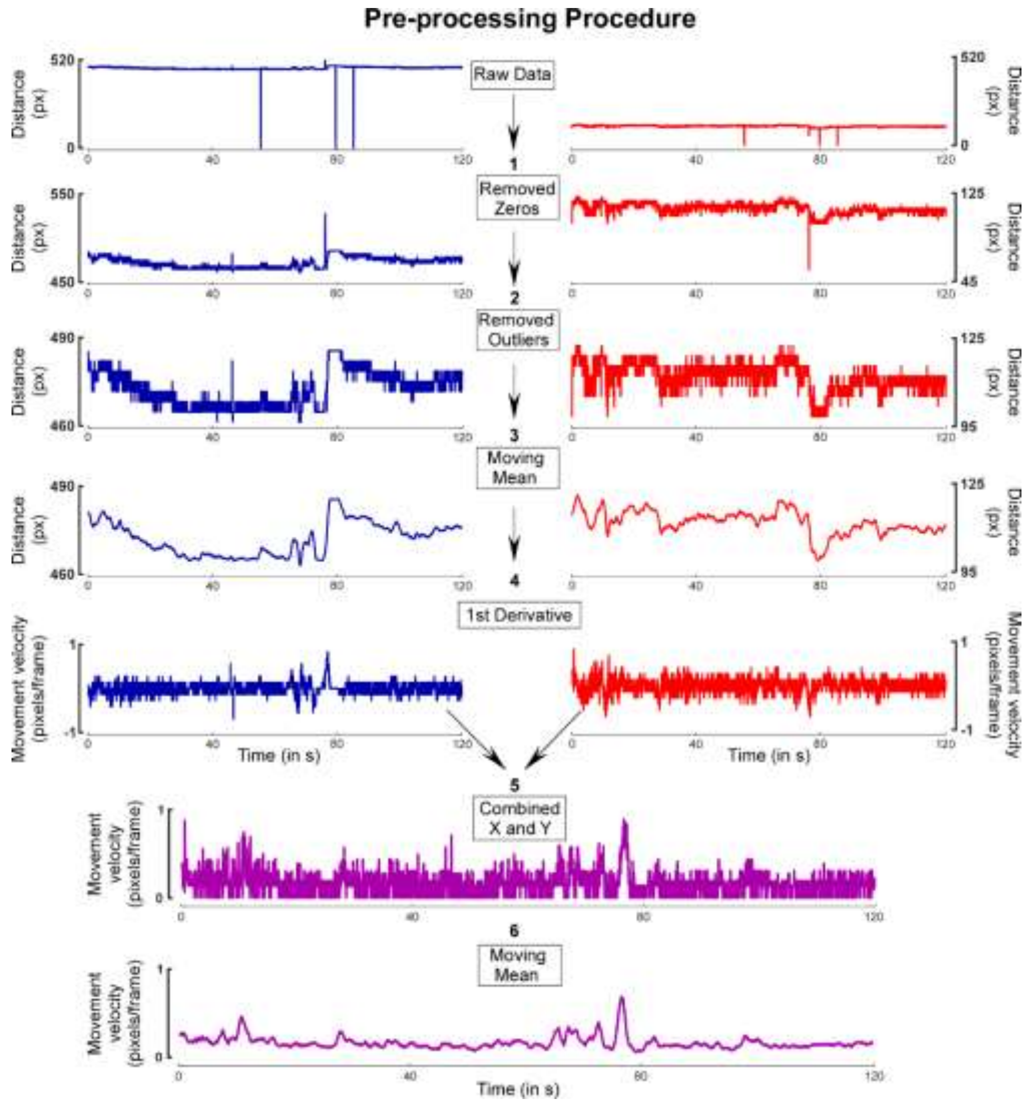

Figure S1. Preprocessing of body landmark time series yielded by OpenPose and Vicon. A visual representation of the preprocessing procedure leading to the computation of movement velocity. The preprocessing procedure first involved the removal of data points where the algorithm was not able to predict body position (zero values) (denoted as step 1 in the figure), and outliers (defined as values exceeding 3 standard deviations from the mean of each trial, step 2). Subsequently, the missing data points in the time series were interpolated using MATLAB's 1D interpolation function (table lookup). The data were then smoothed using a moving mean (window = 1 sec, step 3). To compute an estimate of body movement, we used the first derivative of the positional data along the x and y axes separately (step 4) and then calculated the Euclidean norm of the resulting velocity vectors (step 5). This transformation served to obtain body part displacement over time, irrespective of the spatial direction of the movement (similar to computing movement speed). Finally, a second moving mean (window = 1 sec) (step 6) was performed on the resulting movement time series.

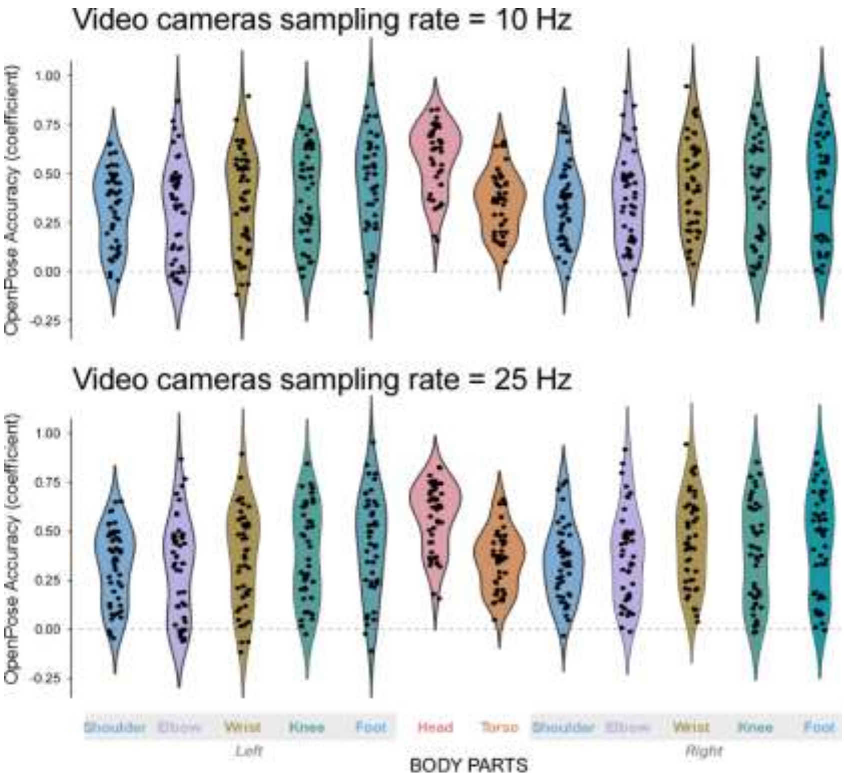

Figure S2. Data downsampling does not change OpenPose accuracy estimates. For control purposes, we repeated the analysis presented in Figure 1B (also shown here, top panel) without downsampling the data from 25 Hz to 10 Hz (bottom panel). The results of this control analysis indicate that decreasing the sampling rate to 10 Hz did not impact upon the accuracy estimates.

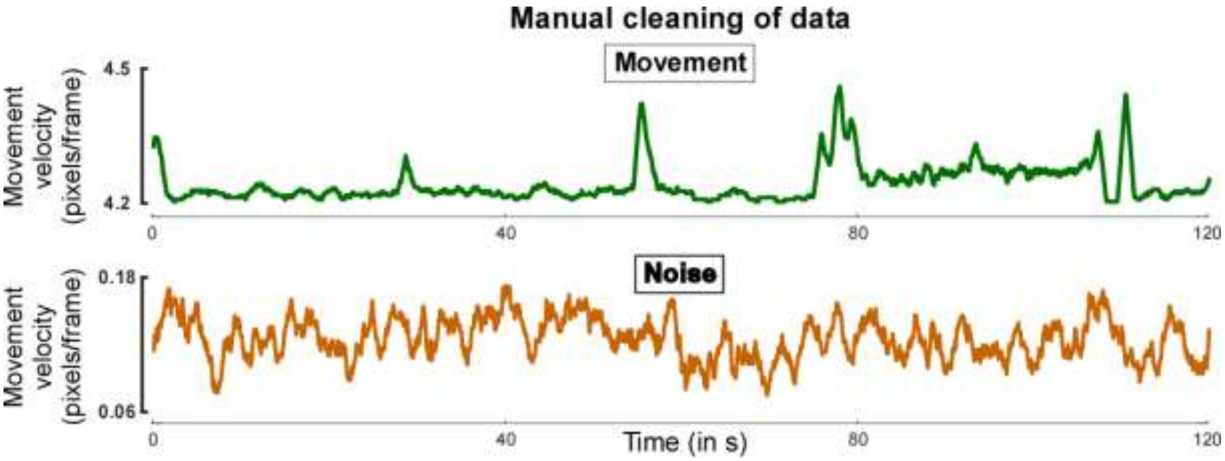

Figure S3. Manual verification of data cleaning procedure for OpenPose body landmarks. The preprocessed body landmark time series from OpenPose were further visually inspected for noise. Each time series was visually compared with its corresponding video recording (body part by body part) to detect trials compromised by artifacts (e.g., high degree of variance). Such trials were removed from further data analysis (2.17% of all data). Example time series for a typical (upper panel) and noisy (lower panel) body landmark are shown in the figure.

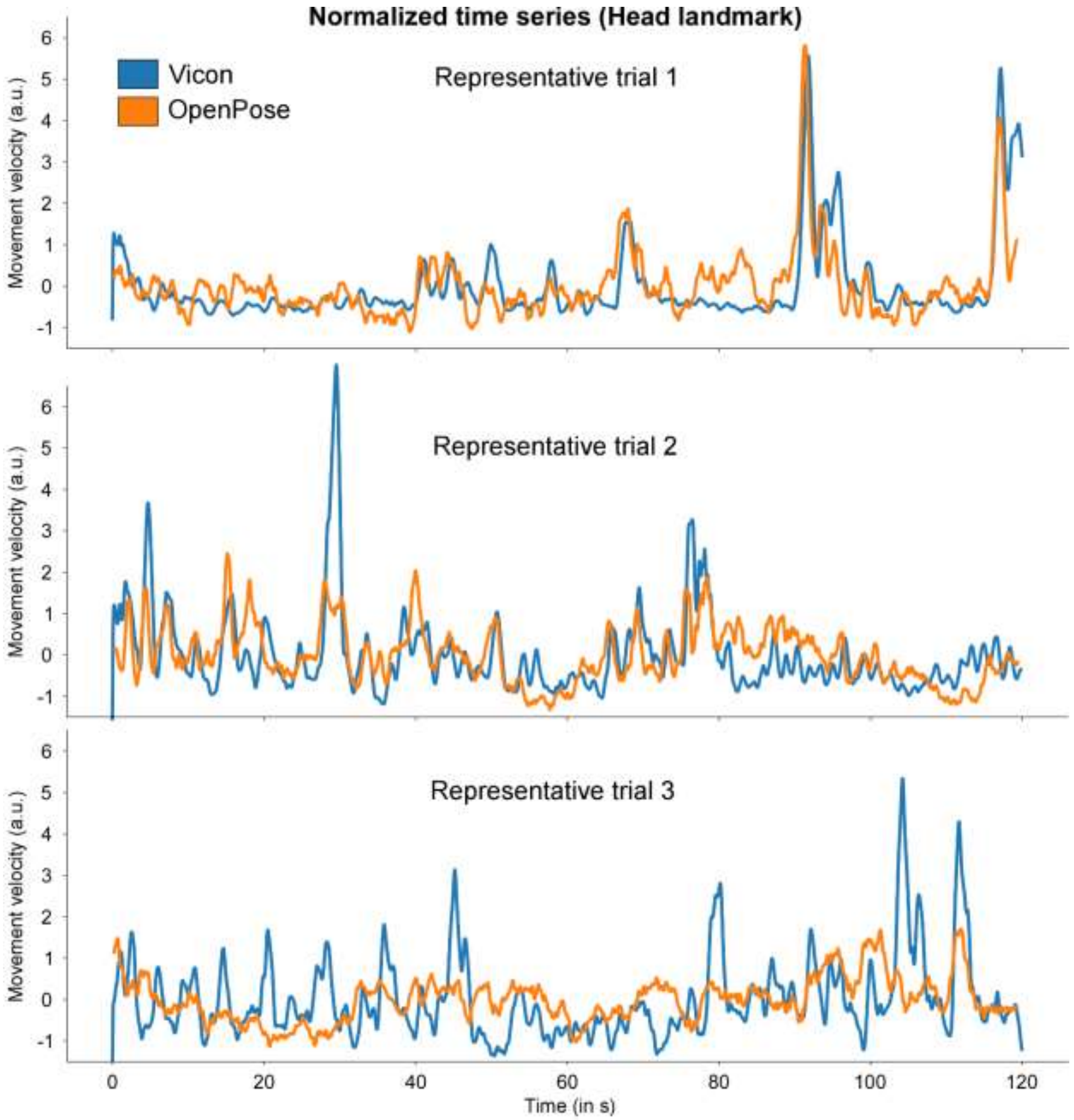

Figure S4. Representative data. Representative velocity time series (Head landmark) from three random trials, following data preprocessing (Figure S1). The velocity time series extracted from Vicon (blue) and OpenPose (orange) datasets were correlated to yield coefficients indexing OpenPose accuracy.

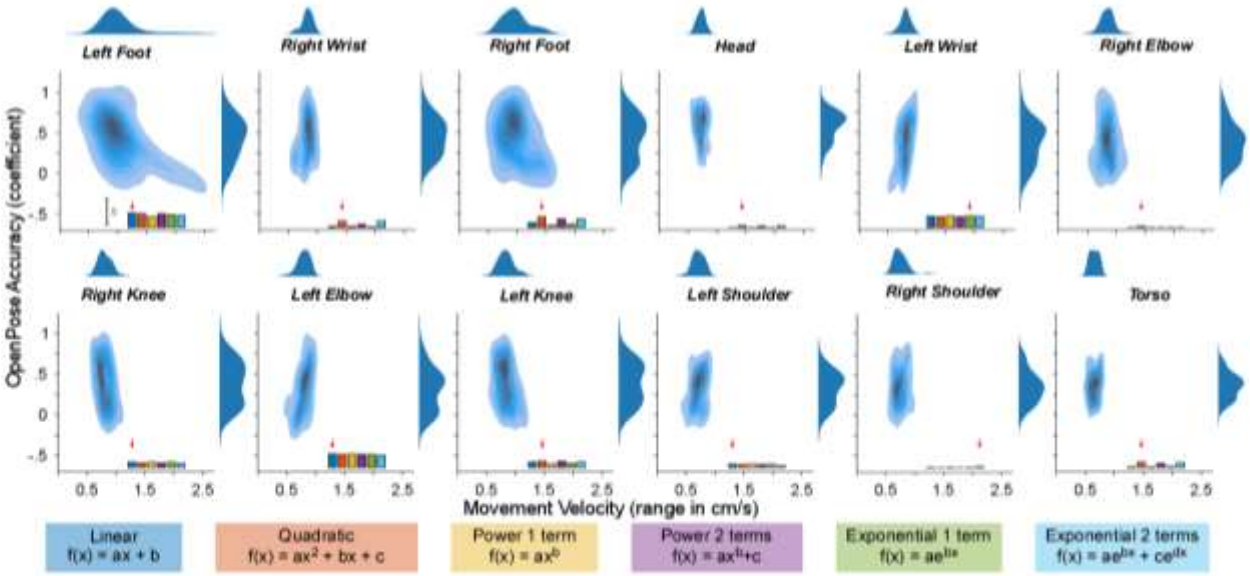

Figure S5. Relationship between OpenPose accuracy and movement velocity. We examined whether movement parameters other than amplitude can predict OpenPose accuracy. To this aim, the analysis presented in Figure 2 was repeated using movement velocity, instead of amplitude, to predict OpenPose accuracy. The results indicate that both movement parameters (amplitude and velocity) can predict OpenPose accuracy, although the best-fitting curve characterizing their relationship is typically different: most often quadratic when velocity is used as a predictor. Such a quadratic relationship might suggest that accuracy is lowest when velocity is either too high or too low.

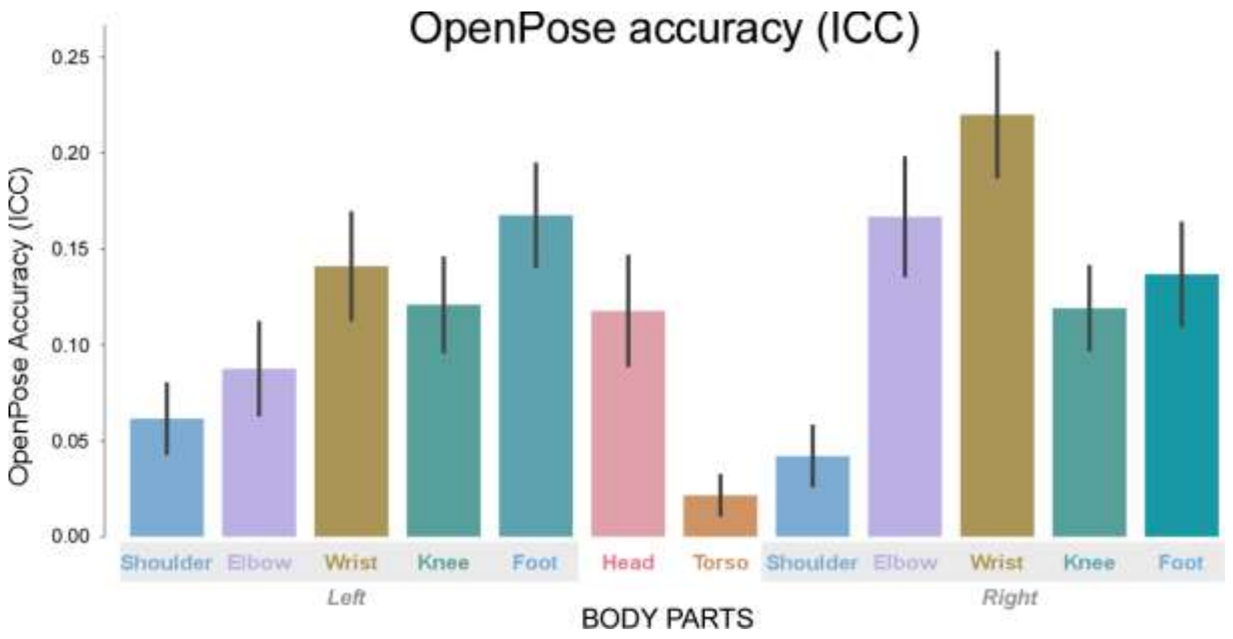

Figure S6. OpenPose accuracy computed using intraclass correlation coefficients. Bar plots indexing OpenPose accuracy, averaged across participants, separately for each body part. Accuracy was estimated using intraclass correlation coefficients (as opposed to Pearson's correlation coefficients, cf. Figure 1) resulting from the correlation between movement velocity time series yielded by OpenPose and Vicon (ground truth). Intraclass correlation (two-way random effects, absolute agreement, single rater/measurement) was computed for each trial, body part, and participant. We then averaged these coefficients for each participant. To estimate the significance of the accuracies, we compared averaged (real) accuracies to those obtained from randomly shuffling data across trials and participants (pseudo accuracies) (note that t-tests vs. zero were not an option in that intraclass correlation coefficients range between 0 and 1). All the 'real' accuracies were significantly higher than the 'pseudo' accuracies ($p<0.05$). Error bars represent one standard error of the mean.
